# Off-Label Use of a Double-Layer Micromesh Carotid Stent for Hybrid Treatment of Popliteal Artery Aneurism Complicated by Chronic Distal Embolization

**DOI:** 10.1155/2021/5546194

**Published:** 2021-06-21

**Authors:** Sorin Barat, Dumitru Casian

**Affiliations:** ^1^Division of Vascular and Endovascular Surgery, Republican Clinical Hospital, 29 Nicolae Testemitanu Str., Chisinau, Moldova; ^2^Department of General Surgery Nr.3, Vascular Surgery Clinic, Nicolae Testemitanu State University of Medicine and Pharmacy, 165 Stefan cel Mare si Sfant Bd., Chisinau, Moldova

## Abstract

We report our initial experience in off-label use of the double-layer micromesh (DLM) Roadsaver® stent for the hybrid treatment of a fusiform popliteal artery aneurism complicated by distal embolization and chronic limb threatening ischemia in a COVID-19-positive young male. A 36-year-old male patient was admitted with chronic limb threatening ischemia of the left lower limb. The duplex ultrasound and computer tomography angiography (CTA) demonstrated a fusiform popliteal artery aneurism with a maximal diameter of 14 mm and distal occlusion of peroneal and both tibial arteries. Urgent hybrid intervention was performed, starting with an open thrombectomy from the distal posterior tibial artery via a retromalleolar access followed by percutaneous deployment of the DLM Roadsaver® stent (Terumo, Tokyo, Japan) for the exclusion of the popliteal artery aneurism. The flow diverting effect was observed immediately with contrast stagnation in the asymmetrical part of the aneurism sac (grade C2 of the O'Kelly-Marotta flow diversion scale). The procedure was uneventful, with the regaining of an adequate foot perfusion and palpable pulse at the posterior tibial artery. On the 2^nd^ postoperative day, the patient was diagnosed with a symptomatic form of COVID-19 infection and transferred to a dedicated facility. At a one-month follow-up, the patient had no symptoms of limb ischemia and CTA showed complete thrombosis of the aneurism sac, absence of endoleaks, and patency of the treated arterial segment. This case demonstrates the possibility of off-label use of the DLM Roadsaver® stent for hybrid treatment of popliteal artery aneurism complicated by distal embolization and critical limb ischemia.

## 1. Introduction

Popliteal artery aneurysms, defined as a focal dilatation with a diameter more than 50% of the reference vessel diameter, account for 85% of all peripheral aneurisms. Complications occur in up to 30% cases of untreated popliteal aneurysms and are associated with a high risk of limb loss. These include aneurism thrombosis and distal embolization, manifested by acute or chronic limb ischemia, compression syndrome, and rupture. The rate of amputation due to runoff vessel occlusion is up to 15% [1, 2].

All symptomatic popliteal artery aneurisms require treatment because of the high incidence of limb loss. Asymptomatic aneurisms with a diameter greater than 2 cm are considered indication for elective aneurism repair. Open repair via a medial or posterior approach represents the standard treatment for large or symptomatic popliteal artery aneurysms and consist in aneurysm exclusion with autologous or prosthetic bypass grafting. Recently, percutaneous implantation of the PTFE-covered stents is considered an alternative approach associated with low perioperative morbidity and mortality [3].

We describe a clinical case of hybrid treatment of complicated small popliteal artery aneurysm by tibial thrombectomy and off-label use of a double-layer micromesh (DLM) stent.

## 2. Case Report

**Figure 1:**
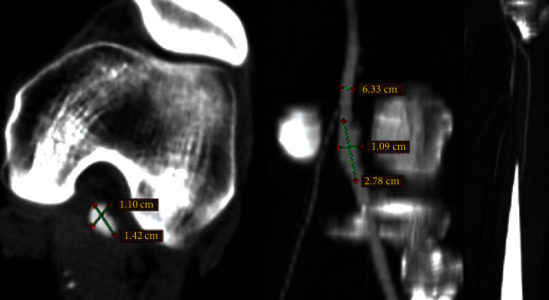
Computer tomography angiography demonstrating fusiform aneurysm of the left popliteal artery with a maximal diameter of 14 mm and distal occlusion of tibial and peroneal arteries.

A 36-year-old male was admitted to the division of vascular surgery with clinical signs of chronic limb threatening ischemia Rutherford grade 4, with an onset of symptoms beyond 3 months. At physical examination, the left foot was hypothermic and pulseless, with a delayed capillary refill. The ankle-brachial pressure index (ABI) was 0.5. The emergency duplex ultrasound and CT angiography demonstrated an eccentric fusiform aneurysm of the left P2 popliteal artery with a maximal diameter of 14 mm, complicated by distal embolic occlusion of all three runoff vessels (Figure 1). The ipsi- and contralateral saphenous veins were considered unsuitable for bypass (<3 mm diameter), based on the results of preoperative duplex ultrasound. The decision was to perform a hybrid lower limb revascularization: open tibial thrombectomy and endovascular repair of the aneurysm. The Roadsaver® DLM carotid stent was selected for percutaneous implantation counting on its superior flexibility, easiness of deployment, and possibility of resheathing. Informed consent was obtained from the patient for off-label use of the carotid stent in the popliteal position.

**Figure 2:**
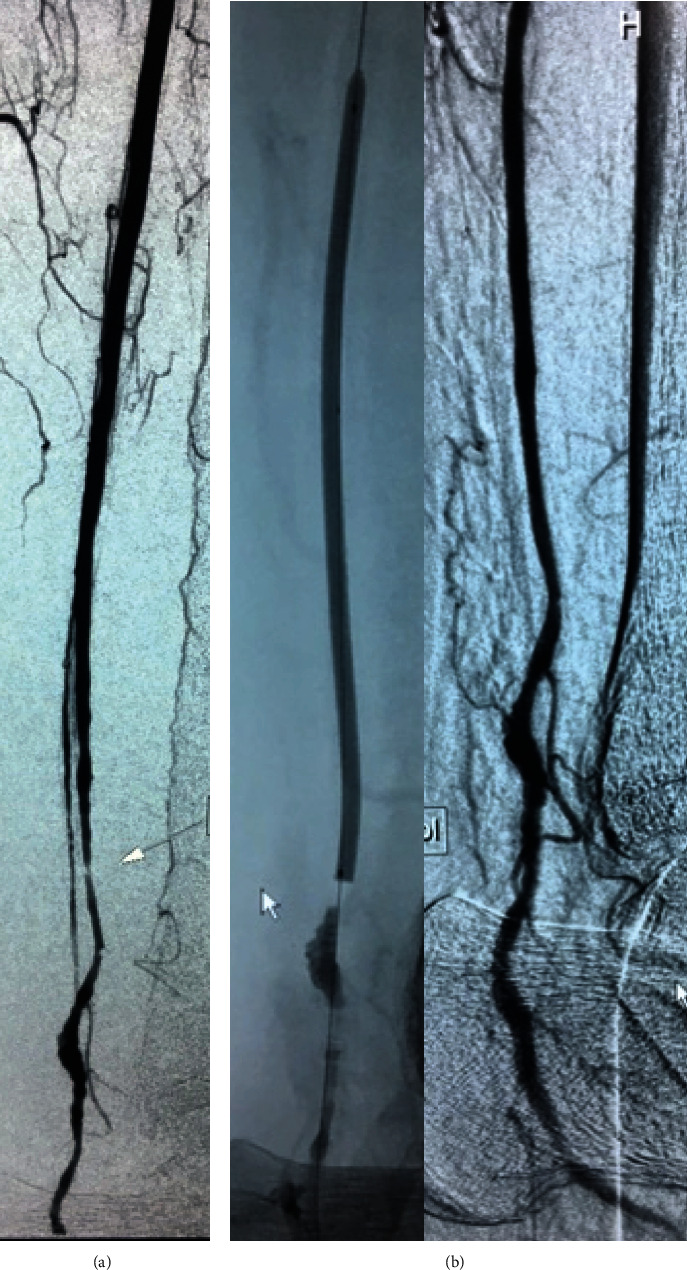
Completion angiography after open thrombectomy: residual stenosis of the posterior tibial artery treated with balloon angioplasty.

**Figure 3:**
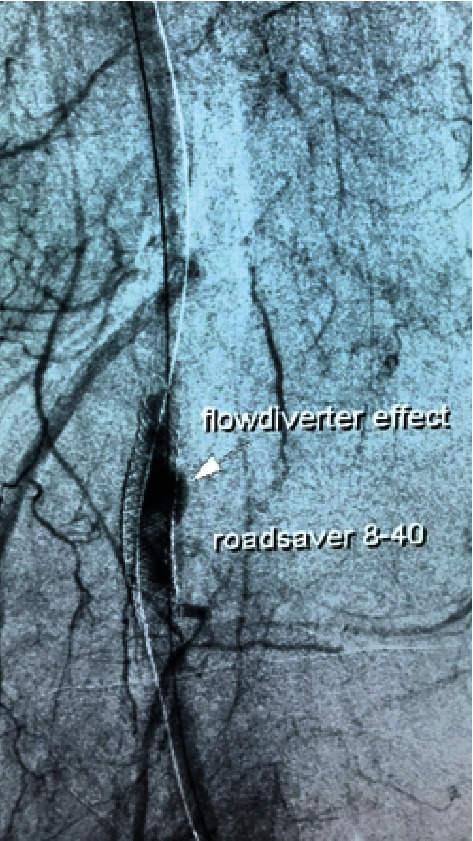
Flow diversion effect after stent implantation: flow stagnation between the aneurism wall and the stent, corresponding to the grade C2 of the O'Kelly-Marotta scale.

During surgery, the posterior tibial artery was dissected via retromalleolar access and direct and indirect thrombectomy was performed, regaining an adequate arterial backflow. The arteriotomy was closed using an autologous venous patch. Completion angiography demonstrated a residual stenosis of the posterior tibial artery, and a balloon angioplasty was performed resulting in a satisfactory runoff (Figure 2). The intervention was continued by percutaneous implantation of an 8-40 mm Roadsaver® carotid stent in the popliteal artery via an antegrade femoral approach. The deployment was uneventful, and the immediate flow diversion effect was encountered, with the presence of the flow stagnation between the aneurism wall and the stent, corresponding to grade C2 of the O'Kelly-Marotta flow diversion scale (Figure 3). Thus, reestablishment of an adequate foot perfusion and palpable pulse on the posterior tibial artery was obtained. On the 2^nd^ postoperative day, the patient had fever and the polymerase chain reaction test of the nasopharyngeal swab confirmed SARS-CoV-2 infection. The patient was diagnosed with a symptomatic form of COVID-19 and transferred to a dedicated facility for isolation and treatment. Taking into account the increased risk of thrombotic complications among patients infected with new coronavirus, triple antithrombotic treatment was initiated during hospitalization (clopidogrel 75 mg, dipyridamole 300 mg, and enoxaparine 60 mg). The patient was discharged on the 14^th^ postoperative day with recommendations to continue dual antiplatelet therapy combined with 10 mg of rivaroxaban per day for one month.

**Figure 4:**
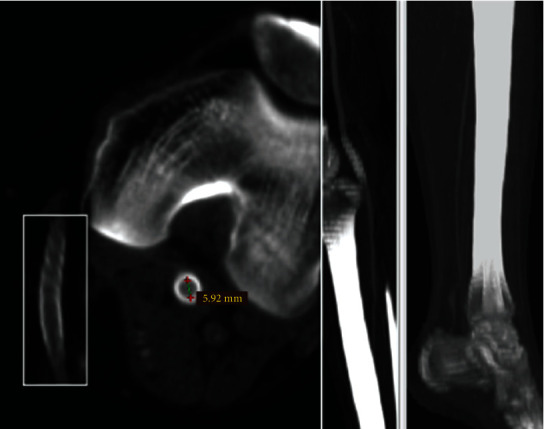
Follow-up CT angiography: complete exclusion of the aneurism and preserved patency of the posterior tibial artery.

At a one-month follow-up, the patient was completely asymptomatic, with palpable pulse at the posterior tibial artery, slightly delayed capillary refill (3 sec), and an ABI of 0.9. The CT angiography showed complete exclusion of the aneurism and preserved patency of the posterior tibial artery (Figure 4).

## 3. Discussion

The primary aim of the intervention for complicated popliteal artery aneurysm is the complete aneurysm exclusion and revascularization of the lower limb. Surgical bypass to the distal popliteal or infrapopliteal arteries using a good-quality saphenous vein is considered a preferential approach. If autologous conduit is not available, the prosthetic conduit can be used, yielding comparable primary and secondary patency [4]. During the last decades, endovascular techniques have gained popularity in popliteal artery aneurysm repair as an alternative to an open surgical approach. In the presented case, we preferred an “endovascular first” approach considering the young age of the patient and the absence of adequate saphenous conduit. In our view, open repair would exclude the possibility of endovascular reintervention if the synthetic graft would have eventually occluded.

Recent studies show that percutaneous implantation of covered stents is a safe and effective technique for the treatment of popliteal artery aneurysms, especially in high-risk patients. The advantages of endovascular treatment include shorter hospital stay and operative time compared to open surgery. Disadvantages include a higher 30-day graft thrombosis rate (9% in the endovascular treatment group vs. 2% in the open surgery group) and a higher 30-day reintervention rate (9% in the endovascular treatment group versus 4% in the open surgical treatment group) [1, 5]. A meta-analysis of 14 studies, reporting 4880 popliteal aneurism repairs, demonstrated better primary patency for open surgery compared to endovascular treatment with no difference in secondary patency at one and three years [6].

A systematic review by Patel et al., regarding the current status of Hemobahn/Viabahn endografts for the treatment of popliteal aneurysms, reported outcomes from 514 PAAs. There was considerable heterogenicity in reporting standards among studies. Pooled primary and secondary patency rates were 69.4% (95% CI 63.3% to 76.2%) and 77.4% (95% CI 70.1% to 85.3%), respectively, at 5 years. Five studies (including only one randomized controlled trial) compared surgical to endovascular repair with no difference in primary patency on evidence synthesis (hazard ratio 1.30, 95% CI 0.79 to 12.14, *p* = 0.189) [7].

Another promising endovascular approach for popliteal artery aneurism repair is the use of uncovered stents with a flow diverting effect.

There are a small number of published studies regarding the use of flow diversion effect in the peripheral arterial circulation, especially in the popliteal arterial segment. In a review, by summarizing the data of 12 published articles, the flow diverters were used for the treatment of extracranial arterial aneurysms in 35 patients. The aneurysms were located in the hepatic (*n* = 12), splenic (*n* = 6), renal (*n* = 5), celiac (*n* = 4), superior mesenteric (*n* = 3), subclavian (*n* = 2), gastroduodenal (*n* = 1), and popliteal (*n* = 1) arteries and in the descending thoracic (*n* = 1), suprarenal (*n* = 1), and infrarenal aorta (*n* = 2). The 30-day mortality was 5.7%. Three stent occlusions occurred, and none of them have clinical consequences. Thirty patients were followed up for a mean interval of 9.2 months. Complete thrombosis of the aneurism sac occurred in 90.6%, and diameter reduction was observed in 81% of the aneurysms. No branch vessel occlusion was observed [8]. Recently, several case reports were published sharing the experience in the use of DLM stents (Roadsaver®, Casper) for treatment of renal, visceral, and supra-aortic extracranial aneurysms and pseudoaneurysms. All authors report technical feasibility and effectiveness of the intervention with complete aneurysm thrombosis at a short-term follow-up [9–12].

The experience of implantation of flow diverting stents for the treatment of popliteal aneurysms is also limited, and results are not uniform. Thakar treated 6 popliteal artery aneurysms using the Cardiatis Multilayer Aneurysm Repair System (MARS). Imaging studies performed after the procedure demonstrated aneurysm exclusion, graft patency, and preservation of branch and runoff arterial flow. There were one symptomatic stent occlusion and one leak through the stent struts into the sac with no branch outflow identified. Two other symptomatic stent occlusions were diagnosed within a 6-week follow-up period, resulting in 3 occlusions among the 6 devices deployed [13]. A similar study performed by Serracino-Inglott showed a primary patency of 50% and secondary patency in only two-thirds of the patients. However, the author states that “…one must note that the patient I managed who ended up with two blocked popliteal MARS had been diagnosed with metastatic lung carcinoma, and it is therefore possible that this may have contributed to a pro-thrombotic state, even though he was on dual antiplatelet therapy” [14].

Ucci et al. reported 23 consecutive patients with 25 popliteal artery aneurysms treated with multilayer flow modulators (Cardiatis). Complete aneurysm sac thrombosis occurred in 92.9% of the cases at 18 months. Freedom from sac enlargement was 100% (with 17 cases of aneurysm sac shrinkage). At 1, 6, 12, and 24 months, the estimated primary patency was 95.7%, 87.3%, 77%, and 70.1%, respectively. At the same intervals, primary-assisted patency was 95.7%, 91.3%, 86%, and 86%, respectively, and secondary patency was 100%, 95.7%, 90.3%, and 90.3%, respectively. The collateral vessel patency was preserved in 72.4%. The authors report one case of stent fracture in an asymptomatic patient [15]. Promising results of percutaneous treatment of popliteal aneurysm are also reported with the use of the nitinol interwoven-wire Supera stent (Abbott Vascular, Illinois). The authors achieved 100% technical success, freedom from sac enlargement and prevention of distal embolization. However, complete sac thrombosis at a one-year follow-up was observed only in 34% of the cases [16].

While uncovered stents seem to be effective in preventing aneurysm expansion and distal embolization, there is a risk of stent occlusion, especially in patients with prothrombotic states. The infection with new coronavirus is associated with a significant rate of arterial and venous thrombosis, and anticoagulation is recommended for all hospitalized patients [17]. However, current evidence regarding the optimal antithrombotic treatment of COVID-19 patients undergoing endovascular interventions is still lacking. Considering low bleeding risk and high cumulative risk of thrombosis (caused by stent implantation, single-vessel runoff, and SARS-CoV-2 infection) in our patient, we decided to use the triple therapy: two antiplatelet agents combined with a direct anticoagulant. The similar approach is recommended by current guidelines for patients with acute coronary syndrome and SARS-CoV-2 infection [18]. During in-hospital treatment, the enoxaparine was used as the anticoagulant of choice in order to prevent possible interactions with antiviral drugs. After discharge, it was replaced with prophylactic dose of rivaroxaban due to the possibility of oral administration. Dipyridamole was used as a component of dual antithrombotic therapy considering its potential benefits in the treatment of SARS-CoV-2 virus infection. Several publications mentioned the ability of this drug to suppress SARS-CoV-2 replication *in vitro* and improve the clinical course of the disease [19, 20].

## 4. Conclusion

The existing body of evidence regarding the use of flow diverting stents for the treatment of popliteal artery aneurisms is scarce, and there are not any previous reports of Roadsaver® carotid stent implantation for this purpose. The authors believe that their initial experience with off-label use of the Roadsaver® stent for the hybrid treatment of complicated popliteal artery aneurisms is promising and can serve as a background for further studies.

## Data Availability

The diagnostic and interventional data used to support the findings of this study are included within the article.
